# Stent thrombosis: a contemporary guide to definitions, risk factors, and management

**DOI:** 10.3389/fcvm.2025.1622235

**Published:** 2025-10-21

**Authors:** Ambre Flowers, Bernard Evenhuis, Benjamin Gabanic, Allison Weiss, Scott Eisenberg, Riyan Siddiqui, Affan Rizwan, Iqra Riaz, Hafeez Ul Hassan Virk, Mahboob Alam, Muzamil Khawaja, Markus Strauss, Chayakrit Krittanawong

**Affiliations:** ^1^ Department of Internal Medicine, Emory University, Atlanta, GA, United States; ^2^ School of Medicine, University of Mississippi Medical Center, Jackson, MS, United States; ^3^ Departmemnt of Internal Medicine, Baylor College of Medicine, Houston, TX, United States; ^4^ Department of Internal Medicine, Mobile Infirmary Medical Center, Mobile, AL, United States; ^5^ Harrington Heart & Vascular Institute, Case Western Reserve University, University Hospitals Cleveland Medical Center, Cleveland, OH, United States; ^6^ Department of Cardiology, Texas Heart Institute and Baylor College of Medicine, Houston, TX, United States; ^7^ Department of Cardiology, Emory University, Atlanta, GA, United States; ^8^ Department of Cardiology I- Coronary and Periphal Vascular Disease, Heart Failure Medicine, University Hospital Muenster, Cardiol, Muenster, Germany; ^9^ HumanX, Delaware, DE, United States

**Keywords:** stent thrombosis, coronary artery disease, percuataneous coronary intervention, coronary stenting, ischaemic heart disease (IHD)

## Abstract

Stent thrombosis remains a major complication following percutaneous coronary intervention, with significant morbidity and mortality implications. Despite advancements in drug-eluting stents and optimized pharmacotherapy, real-world registry data indicate that definite or probable stent thrombosis occurs in approximately 0.5% of percutaneous coronary intervention cases, with a 30-day mortality rate approaching 25% and a long-term risk exceeding 30% at 10 years. Stent thrombosis is classified based on timing into acute, subacute, late, and very late thrombosis, with subacute and very late stent thrombosis being the most prevalent. Clinical consequences include myocardial infarction, emergent revascularization, and heightened cardiovascular risk, necessitating timely recognition and intervention. Risk factors include patient characteristics, procedural variables, and lesion complexity, with recurrent stent thrombosis remaining a notable concern. This review explores the definitions, classifications, pathophysiology, and risk factors for stent thrombosis while discussing current strategies for prevention and management. Additionally, advancements in stent technology and pharmacologic interventions are examined, underscoring the need for a multidisciplinary approach to mitigate stent thrombosis incidence and improve patient outcomes.

## Introduction

Stent thrombosis (ST) remains one of the most significant complications following percutaneous coronary intervention (PCI), carrying significant implications for patient outcomes. Although its overall incidence has decreased in the era of contemporary drug-eluting stents (DES) and optimized pharmacotherapy, ST continues to be associated with substantial morbidity and mortality. Recent real-world registry data show that definite or probable ST occurs in approximately 0.5% of PCI cases, and when it does occur, nearly one in four patients may die within 30 days (1). Long-term data have demonstrated that mortality risk persists well beyond the initial event, rising to over 30% at 10 years (2). The clinical consequences of ST typically include myocardial infarction, emergent revascularization, and a heightened risk of death, emphasizing the need for timely recognition and appropriate management strategies. These findings underscore why, despite modern advancements, a thorough understanding of ST and its clinical course remains essential for guiding effective patient care. The American College of Cardiology (ACC) and American Heart Association (AHA) define ST as the formation of a thrombus within a stent that leads to occlusion of the stented segment. Definite ST requires angiographic confirmation or autopsy findings, while probable ST is suspected in cases of unexplained death within 30 days of stent placement or myocardial infarction attributed to the stented vessel. Possible ST is a diagnosis of exclusion in patients who die more than 30 days post-implantation without another identifiable cause (3).

**Figure 1:**
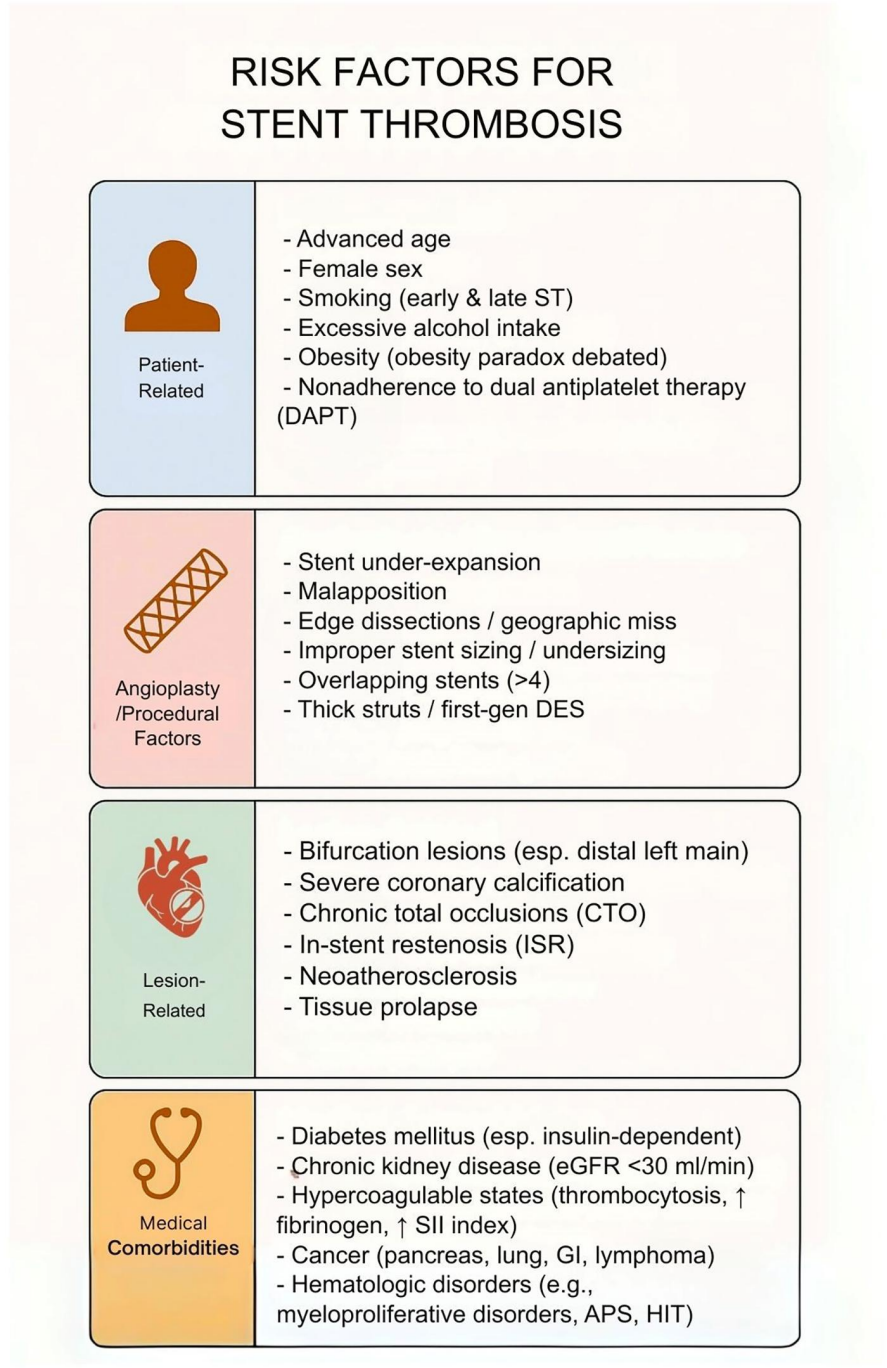
Risk factors for stent thrombosis. This table illustrates that risk factors for stent thrombosis are multifactorial, spanning patient-related, procedural, lesion-related, and comorbid conditions. While nonmodifiable factors such as age and sex contribute, modifiable risks—including smoking, obesity, nonadherence to dual antiplatelet therapy, stent under-expansion, and lesion complexity—play a central role. Recognition of these domains is critical for tailoring procedural strategies, optimizing pharmacotherapy, and reducing early and late stent thrombosis.

ST is classified into four categories based on timing: acute (within 24 h of stent implantation), subacute (24 h to 30 days), late (31 days to one year), and very late (beyond one year) thrombosis (3). Studies have shown variable prevalence rates of ST based on timing. Tariq et al. (4) reported an overall ST rate of 5.8% in a cohort of 569 patients followed for 30 days post-stent implantation, with acute ST accounting for 0.5% and subacute ST for 5.3% (4). The PRESTIGE registry, which utilized optical coherence tomography (OCT), found the prevalence of acute/subacute, late, and very late ST to be 6.1%, 28.6%, and 71.4%, respectively (5). Similarly, the PESTO French Registry identified very late ST as the most common form (75%), followed by subacute (15%), late (6%), and acute ST (4%) (6). Mohamed et al. (7) found that early ST accounted for 52.6% of cases, late ST for 12.0%, and very late ST for 35.4% (7). These studies highlight the variability in ST prevalence based on timing, with subacute and very late ST appearing most frequently. Recognizing these patterns is important for clinical risk stratification and guiding preventive efforts.

The clinical implications of ST are profound. Yang et al. (8) highlighted that early ST is associated with an increased risk of major adverse cardiovascular and cerebrovascular events (MACCE) (8). Tovar Forero et al. (9) reported a 43.7% major adverse cardiovascular event (MACE) rate within 60 days of ST, with 19.5% experiencing cardiac death and 17.9% suffering myocardial infarction (9). Ohno et al. (10) similarly found that ST leads to higher in-hospital mortality and increased cardiovascular complications (10). These findings highlight the importance of close follow-up and proactive treatment during the early phase following ST.

The long-term clinical implications of ST remain concerning, as mortality rates do not decrease over time. Ishihara et al. (2) found that mortality rates increased from 14.6% at 1 year to 33.8% at 10 years post-ST. Additionally, patients with a history of hemodialysis, culprit lesions in the left main trunk or left coronary artery, or elevated creatine kinase levels were at increased risk of mortality (2). Yang et al. (8) meta-analysis further demonstrated that patients with late and very late ST face significantly higher mortality rates at various time points, including in-hospital (*P* = 0.004), 30-day (*P* < 0.00001), 1-year (*P* < 0.00001), and long-term mortality (*P* = 0.04) (doi:10.1007/s11239-020-02184-7), underscoring the persistent risk even years after the initial event (8). Furthermore, the likelihood of recurrent ST is not negligible; Tovar Forero et al. (9) reported a restenosis rate of 12.1% (9). Given these long-term risks, ongoing surveillance and tailored secondary prevention strategies are essential for optimizing patient outcomes.

While the scope of this review primarily focuses on ST in coronary arteries, it is important to mention that ST in peripheral arteries also results in significant morbidity. The rate of ST in non-coronary vessels varies by vascular territory, stent type, and lesion complexity but is generally significantly higher than in coronary arteries. Large cohort studies reveal a ST rate of 6.1% in th e aortoiliac and femoropopliteal arteries after one year (11) and up to 13.4% in the superficial femoral artery at 5-years (12). Patients who experience stent thrombosis have a markedly increased risk of major adverse limb events, with hazard ratios approaching 5 for events such as repeat revascularization, major amputation, or persistent ischemia within 12 months.

ST remains a serious complication of PCI with significant implications for patient outcomes. This review will explore the definitions, classifications, risk factors, and mechanisms underlying ST, as well as current management and prevention strategies. We will examine how patient characteristics, procedural factors, and lesion complexity contribute to thrombosis risk and discuss the role of pharmacological and interventional therapies. Finally, we will highlight advancements in stent technology and future directions in the field, emphasizing the importance of a multidisciplinary approach to improving patient care and reducing ST incidence.

## Risk factors

Risk factors for stent thrombosis are multifactorial, spanning patient characteristics, procedural variables, lesion complexity, and medical comorbidities (Figure 1). Advanced age, female sex, smoking, and obesity have been linked to increased stent thrombosis risk, though an “obesity paradox” is noted in literature with mixed associations. Nonadherence to DAPT is a major risk factor in the early post-PCI period. Procedural factors including stent under-expansion, malapposition, and edge dissections are important risk factors, prompting a use of intravascular imaging for procedural optimization. Lesion characteristics such as bifurcations, calcifications, chronic total occlusions, and in-stent restenosis, increase stent thrombosis risk. Finally, chronic conditions such as diabetes, chronic kidney disease, and hypercoagulable states increase stent thrombosis risk.

## Patient factors

Age has been reported as an independent predictor of stent thrombosis, as older patients exhibit more progressive vascular changes and thereby increased coronary artery calcification (13, 14). Coronary artery calcification is a major contributor to stent thrombosis and affects more than 90% of men and 67% of women older than 70 years of age (13). Furthermore, over one-third of all percutaneous coronary interventions occur in patients older than 75 years of age which further heightens the prevalence of stent thrombosis within this cohort. Across genders, female sex is associated with a higher risk of stent thrombosis owing to differences in vessel size, endothelial function, and hormonal predisposition toward thrombosis (15). Smoking is a well-documented independent risk factor for stent thrombosis with reported associations for both early and late events (14, 16–19). In smokers, thrombosis risk is heightened through endothelial dysfunction and platelet reactivity (20). Consequently, smokers exhibit a greater risk of stent thrombosis and generally worse outcomes (19). Risk is compounded in smokers with first-generation DES implants, which may further inhibit endothelial cell proliferation and thereby impair vascular healing in this cohort (20). However, associations are reported between smoking and both early and late stent thrombosis in both first- and second-generation DES (21, 22). In addition to smoking, excessive alcohol consumption has also been reported in association with heightened risk of stent thrombosis (23).

Obesity has been reported as an independent predictor of stent thrombosis, with an estimated 2-fold increase in risk (24). Moreover, overweight (BMI 25-29.9) patients are reported to have an increased 10-year risk of stent thrombosis compared to patients with normal BMI (25). However, conflicting evidence has led to the characterization of an “obesity paradox”, wherein an elevated BMI is often not found to be associated with significantly increased risk or may even be protective of poor ischemic outcomes (26). This phenomenon may be a result of several factors, including more aggressive medical therapy and screening or obese cohorts exhibiting a younger age overall with fewer frailties and better cardiac reserve (27). Obesity may contribute to stent thrombosis risk through platelet hyperactivation and accelerated aggregation, endothelial dysfunction, and chronic inflammation (28). Obese patients may also exhibit antiplatelet resistance, leading to suboptimal platelet inhibition with some clinicians adopting a weight-based dose adjustment strategy (19, 29). Nonadherence to DAPT also is a leading risk factor for stent thrombosis. Termination of DAPT prematurely, either by way of patient self-discontinuation, bleeding diathesis, or periprocedural planning, is one of the strongest and most-reported predictors of stent thrombosis, especially within the 30 day post-PCI period (16, 19, 21, 23, 30). This association is particularly strong with early interruption of clopidogrel, as the median time for thrombosis following clopidogrel discontinuation is approximately 9 days within the first 6 months post-implantation and 104 days beyond 6 months (22, 31). Furthermore, while shorter DAPT durations (3–6 months) are associated with heightened risk of stent thrombosis in ACS patients, evidence for heightened stent thrombosis risk in stable CAD patients is less established (19, 32).

Regional and socioeconomic differences—such as access to contemporary stents, advanced intracoronary imaging, and DAPT significantly impact stent thrombosis rates and outcomes. Multiple large-scale cohort studies and meta-analyses have demonstrated that lower socioeconomic status (SES) measured by income, education, employment, or area deprivation indices—is associated with higher rates of major adverse cardiac events (MACE), including stent thrombosis, recurrent myocardial infarction, and increased long-term mortality after PCI (33, 34). These associations persist even after adjustment for baseline clinical risk factors, though some attenuation occurs, suggesting that both SES and comorbidities contribute independently to adverse outcomes (33). Patients from lower SES backgrounds are less likely to receive contemporary drug-eluting stents, have lower adherence to guideline-recommended dual antiplatelet therapy, and experience higher rates of repeat revascularization and recurrent myocardial infarction, all of which are established risk factors for stent thrombosis (34, 35). Community-level deprivation, as measured by indices such as the Area Deprivation Index, is also linked to higher short-term mortality and readmission rates after PCI, further supporting the role of socioeconomic context in influencing stent-related outcomes (36). These findings highlight the need for targeted interventions to address disparities in access, adherence, and follow-up care to reduce stent thrombosis risk in socioeconomically disadvantaged populations.

## Angioplasty-related factors

Stent under-expansion is a strong predictor of stent thrombosis and increases the relative risk by approximately 13 times (37). This phenomenon, wherein a stent does not expand to the intended diameter after deployment, is more frequently observed in DES than BMS and is present in an estimated 26% of early stent thrombosis patients (20, 21). Stent under-expansion may occur due to undersizing of stents, low deployment pressures, or in the setting of heavily calcified lesions (30). This represents a modifiable risk factor, as use of intravascular ultrasound and optical coherence tomography can optimize stent expansion, e.g., with use of minimum stent area (MSA) of >5.0 mm^2^ (IVUS) or >4.5 mm^2^ (OCT) as an expansion target or with OCT features such as calcium arc ≥180°, calcium thickness ≥0.5 mm, and lesion length >5 mm as predictors of under-expansion (13, 30). Notably, IVUS-measured MSA following PCA has been reported as a predictor of 2-year stent thrombosis risk (15).Stent geometry also plays a role, as uncovered stent struts disrupt early endothelialization and enable platelet attachment and, by extension, thrombus formation (38). Thicker struts may promote flow recirculation and low ESS, ultimately favoring thrombus formation with higher reported rates of stent thrombosis (39, 40). Thinner struts are reported to be associated with lower rates of stent thrombosis (32). Malapposition is a key mechanism contributing to stent thrombosis that is more commonly seen in VLST in DES compared to BMS (20, 23). This phenomenon, in which part of the implanted stent lacks contact with the surrounding endothelium, may lead to altered local hemodynamic and increased blood viscosity, thereby promoting late stent thrombosis (21, 30). Malapposition is estimated to be present in as much as 18.1% of stent thrombosis patients (15). Edge dissections, wherein stent implantation leads to tearing of the arterial wall at the proximal or distal margin of the stent, has been reported as a procedural risk factor for early stent thrombosis and a major failure mechanism in very late stent thrombosis (20, 22). Expert consensus reports that geographic miss, edge dissections, and intramural hematomas may be strong procedural predictors of early stent thrombosis (30).

Other procedural factors, such as improper stent sizing (i.e., diameter under sizing) leading to incomplete lesion coverage are important risk factors for stent thrombosis (23). Stent under sizing is of particular importance in DES and BRS (40). In an effort to modify this risk, IVUS and OCT have been employed which may lead to a reduction in stent thrombosis risk, particularly in ACS and complex lesions (21, 22). Furthermore, the use of 4+ stents during PCI has been identified as a risk factor for stent thrombosis (17). Overlapping of stents is more frequently observed in stent thrombosis cases compared to non-thrombosis controls (36.0% vs. 24.7%) (41). Finally, first-generation DES generally have a higher risk of early stent thrombosis compared to second-generation DES, owing to polymer coating differences and improved drug release kinetics (31).

## Lesion-related factors

Coronary bifurcations are particularly prone to thrombosis due to altered hemodynamics and unbalanced wall shear stress, especially in cases involving stent underexpansion and malapposition (14, 20). Studies involving the REAL-ST and KoST registries reported that bifurcation lesions are significantly more frequent in stent thrombosis patients than in non-thrombosis controls (45.5% vs. 36.5%) (41, 42). Stenting across major side branches is thought to alter flow dynamics and lead to increased platelet activation, leading to increased risk of stent thrombosis, especially within distal left main bifurcation lesions (14, 18, 30). Addressing bifurcations lesions via a two-stent strategy has been reported as a procedural risk factor for early ST (22). Alternative strategies involving overlapping stents are also prone to underexpansion and malapposition (30). Severe coronary calcification leads to stent under-expansion and decreases stent symmetry resulting in malapposition, with suboptimal stent deployment reported in 31%–58% of calcified lesions (30). This ultimately increases risk of stent thrombosis (20, 23, 43). Severe calcification has been estimated to be present in 52.8% of late stent thrombosis cases compared to 14.6% of non-thrombosis controls (41). Calcified lesions generally carry a poorer overall prognosis following PCI, which highlights the importance of intraprocedural lesion preparation and stent deployment in these patients (44). Chronic Total Occlusions (CTOs) also feature higher rates of stent malapposition and under-expansion, leading to increased risk of stent thrombosis (20). CTO's are estimated to be present in 8.3% of stent thrombosis cases vs. 3.3% of non-thrombosis controls (15). Residual plaque burden following intervention in CTO's may contribute to thrombosis formation (18). Tissue prolapse, involving luminal plaque extrusion into the implanted stent, has also been reported as an OCT feature with predictive value of early stent thrombosis in CTO lesions (42). Finally, CTO's are independent predictors of in-stent restenosis, which may further compound risk of stent thrombosis (30). In-Stent Restenosis (ISR), involving vascular smooth muscle cell proliferation into stent struts, also represents an important risk factor of stent thrombosis by way of secondary platelet rupture and thrombus formation (20, 23). ISR is present in an estimated 14.6% of stent thrombosis cases vs. 9.6% of controls (15, 41). ISR is also a known predictor of very late stent thrombosis, especially within second generation DES (42). In cases involving stent under-expansion, this procedural risk factor may contribute to neointimal hyperplasia, accelerating ISR and compounding stent thrombosis risk (44). Finally, neo-atherosclerosis, wherein new atherosclerotic plaques form within the neointima of implanted stents, is a frequent mechanism underlying late and very late stent thrombosis (20). This phenomenon is more common in BMS than in DES, which may contribute to overall heightened stent thrombosis risk in cases involving BMS (20).

## Medical comorbidities

Diabetes mellitus is a well-documented predictor for early, late, and very late stent thrombosis following PCI, and insulin dependence is particularly associated with early stent thrombosis (16, 17, 21–23, 31, 39, 41, 42). Diabetes mellitus increases the risk of stent thrombosis via various mechanisms. Firstly, diabetes is associated with increased platelet aggregation, endothelial dysfunction, and a prothrombotic state, all of which contribute directly to stent thrombosis pathophysiology (19, 20). Secondly, diabetes mellitus accelerates atherosclerosis and in-stent neoatherosclerosis, which are key risk factors of very late stent thrombosis (45), Finally, diabetes mellitus leads to reduced arterial healing, increasing stent strut exposure (19). Consequently, diabetes mellitus is associated with higher rates of late stent thrombosis following PCI, with reported rates of 1.7% vs. 0.9% in the E-FIVE and BIOSCIENCE trials (HR = 1.95, *P* = 0.13), 3% vs. 1.1% in the Western Denmark Heart Registry (RR = 2.56), and an overall OR of 1.95 in a meta-analysis of 18,910 patients (19). In pursuit of avenues to mitigate this risk, polymer-free DES have been identified as a potential solution, with some evidence suggesting a reduction in very late stent thrombosis risk compared to conventional stents (20).

Both chronic kidney disease and end-stage renal disease independently predict stent thrombosis (14, 16, 23). An eGFR < 30 ml/min/m^2^ has been reported to be associated with both late and very late stent thrombosis (22, 31). CKD may contribute to stent thrombosis risk by promoting arteriosclerosis progression, altered platelet function, systemic inflammation, and a prothrombotic state (19, 20, 45). Furthermore, physicians are more likely to prematurely discontinue DAPT in CKD patients due to an underlying bleeding diathesis which may inadvertently increase stent thrombosis risk (19).

Hypercoagulable states and systemic inflammation, by promoting endothelial damage, are key contributors of stent thrombosis (17). This phenomenon is illustrated through multiple studies evaluating proinflammatory and hypercoagulability biomarkers in stent thrombosis. One retrospective study evaluating the systemic immune-inflammation index found that a value ≥636 is an independent predictor of stent thrombosis (17). Elevated fibrinogen levels have also been observed to a higher degree in patients with very late stent thrombosis relative to controls (45). Thrombocytosis >400 K/ml was estimated to be present in 21.2% of early stent thrombosis patients compared to 7.8% in controls (16, 21). Measures of platelet reactivity are reported to be higher in patients with early stent thrombosis vs. controls (31). Hypercoagulable and proinflammatory states are thought to contribute to thrombosis via the contact activation pathway (46).

Consequently, cancer and thrombophilic hematologic disorders are important risk factors for stent thrombosis. Cancer is reported to be a risk factor for both early and late stent thrombosis, with pancreatic, lung, gastrointestinal cancers, and lymphomas exhibiting particularly increased risks for thrombotic events post-PCI (47, 48). Many chemotherapies used to treat cancer further heighten this risk due to prothrombotic properties (19). Further, myeloproliferative disorders result in aberrant blood counts, leading to a prothrombotic state and increasing risk of stent thrombosis (47). Finally, thrombophilic hematologic disorders such as antiphospholipid syndrome and heparin-induced thrombocytopenia lead to excessive platelet activation and impaired anticoagulation balance, resulting in a heightened risk of stent thrombosis (47). Individualized antithrombotic strategies must therefore be considered in patients with malignancy or other hypercoagulable states (20).

## Risk factors and timing

Importantly, the predominant risk factors vary depending on timing. The most common causes of acute stent thrombosis are stent underexpansion and edge dissection along with inadequate antiplatelet therapy initiation (30, 49). Subacute stent thrombosis is most commonly caused by premature discontinuation or inadequate response to dual antiplatelet therapy (DAPT) (30, 50, 51). Late stent thrombosis tends to be related to impaired neointimal healing, which is often due to delayed endothelialization, stent malapposition, or stenting across complex lesions (e.g., bifurcations, overlapping stents) (30). Very late stent thrombosis can be caused by stent malapposition, neoatherosclerosis, and uncovered strut (6, 30). These observations highlight the need for vigilance across all phases after PCI, with tailored strategies to minimize stent thrombosis risk based on patient profile, lesion characteristics, and timing of the event.

## Pathophysiology

The pathophysiology of stent-related thrombosis involves complex interactions between stent materials, drug coatings, and the vascular healing response. Stent materials can induce platelet adhesion and activation, leading to thrombus formation. First generation stents such as paclitaxel-eluting stents (PES), contain a polymeric surface that causes a higher degree of platelet activation and deposition compared to bare metal stents (52) Granada et al. observed that the polymeric surface of the PES induce a higher degree of platelet activation and deposition compared to the BMS surface; however, this is not associated with thrombus formation. The mechanism of this is mediated by increased p-selectin expression and platelet-monocyte complex formation. Cobalt-chromium (CoCr), a second-generation DES, significantly induced thrombin generation by the contact and activation of platelets, as demonstrated by Ollivier et al. (53). Drug-eluting stents can further contribute to thrombogenic risk by impairing re-endothelialization, thereby prolonging the exposure of the stent surface to circulating platelets. Sirolimus and paclitaxel, used in first-generation DES, inhibit vascular smooth muscle cell proliferation which delays endothelial healing. This delayed healing is implicated in late and very late stent thrombosis due to prolonged stent exposure (54). CoCr surfaces can trigger leukocyte adhesion, which acts as a scaffold for neutrophil and endothelial cell monolayer formation. This newly formed endothelial phenotype is dysfunctional, impairing effective healing and fostering a pro-inflammatory, pro-thrombotic environment (53).

## Role of mechanical and hemodynamic factors

There are three major hemodynamic factors contributing to stent thrombosis: endothelial shear stress (ESS), stent positioning, and stent design. ESS, defined as the tangential force exerted by blood flow on the endothelial surface, is influenced by flow velocity and arterial geometry. When a stent is implanted, it alters this geometry, introducing local flow disturbances and changes in ESS. These regions of low or oscillatory ESS promote platelet adhesion and activation, fostering thrombus formation and local inflammation. Implantation of a stent into an artery imposes geometric changes to flow, introducing shearing stress. This ESS promotes platelet adhesion and activation, which leads to thrombus formation. This induces inflammation, endothelial growth factors such as PDGF, VEGF, and ET-1, which promotes neointimal hyperplasia and endothelialization, creating a pro-thrombotic environment (55). Human studies have revealed an inverse relationship between ESS and extent of ISR after BMS implantation. In DES, ISR occurred more extensively in low-ESS regions after sirolimus-eluting and paclitaxel-eluting stent implantations (56). Together, these factors highlight how mechanical and hemodynamic factors create a pro-thrombotic microenvironment that can persist even with advanced stent technologies.

Stent mispositioning plays a significant role in altering local hemodynamics and increasing the risk of IST. Specifically, SM can lead to regions of ESS, which promote platelet adhesion and thrombus formation. Contributing factors to SM include procedural issues such as stent under sizing or under-expansion, plaque-related mechanisms like positive vessel remodeling, and device-related factors such as delayed endothelialization (57). Protruding struts create localized flow disturbances, acting as a foreign body that disrupts laminar flow and facilitates a procoagulable environment. The extent of stent strut detachment has been shown to correlate strongly with thrombus burden. Qu et al. used computational modeling to demonstrate that shear stress induced by SM is a critical driver of thrombosis**,** with greater detachment distances associated with increased thrombus formation (58).

Stent design characteristics further influence thrombogenic potential. Strut thickness and geometry play pivotal roles in modulating blood flow and shear stress. Thicker struts and less streamlined designs disturb flow more significantly, increasing the likelihood of thrombus formation. Clinical studies have shown that thick-strutted stents (>162 µm) are approximately 1.5 times more thrombogenic than their otherwise identical thin-strutted counterparts (≈81 µm) (59, 60). In contrast, thin-strutted stents maintain more favorable hemodynamic conditions and are associated with a reduced risk of thrombosis. Together, these findings highlight how mechanical and hemodynamic factors create a pro-thrombotic microenvironment that can persist even with advanced stent technologies.

## Evidence from randomized clinical trials

### DAPT selection and duration

The DAPT Trial was an international clinical trial to examine the risks and benefits of dual anti-platelet therapy beyond 1 year after placement of a drug-eluting stent as compared with aspirin therapy alone. The DAPT trial had strict inclusion criteria, as only those free from MACE) MACCE), stent thrombosis, repeat revascularization, and moderate or severe bleeding, and who were adherent to DAPT were randomized to either continued thienopyridine or placebo in addition to aspirin for an additional 18 months. This study found that extending DAPT beyond 1 year significantly reduced the risks of ischemic events, such as stent thrombosis, major adverse cardiovascular events, and cerebrovascular events, but was associated with a higher risk of bleeding. The reduction in risk of ischemic events was consistent across stent type. However, this study was limited by selection bias due to exlusion of patients with early events or nonadherence. Furthermore, the trial's run-in period may have led to a healthier, more adherent cohort being randomized, which could underestimate real-world risks and benefit (61). In determining the agent for DAPT, the TRITON-TIMI 38 compared prasugrel with clopidogrel for anticoagulation in adults with moderate to high-risk ACS who were scheduled for PCI. This study found reduction of cardiovascular death, MI, stroke, at a median follow-up of 14.5 months in prasugrel users compared with clopidogrel. This aligned with the information that prasugrel is a greater platelet inhibitor compared with clopidogrel. Although the risk of major and fatal bleeding events was greater with prasugrel, composite clinical benefit appears to favor prasugrel. One exception is noted for patients with a history of cerebrovascular events, for whom the clinical profile of clopidogrel is more favorable. One major downside to this study was that a loading dose of 300 mg of clopidogrel was used, which is now considered suboptimal compared to the 600 mg dose used in contemporary practice. This study cannot be applied to patients who were medicaly managed for ACS or those undergoing elective PCI (62). Meta-analysis performed by Palmerini et al. comparing extended-duration DAPT revealed that shorter DAPT was associated with lower all-cause mortality compared with longer DAPT. Patients in 6-month or 1-year DAPT groups had higher risk of myocardial infarction and stent thrombosis but lower risk of mortality compared with patients treated with DAPT for longer than 1 year. Increased non-cardiovascular mortality (such as higher risk of bleeding) may offset the reduction in cardiac mortality (63).

### Procedural techniques

Appropriate stent placement can influence hemodynamic factors and shear stress, increasing the risk of thrombosis. The ADAPT-DES study was a large, prospective, multicenter registry of 9,961 patients that examined whether intravascular ultrasound (IVUS) guidance would improve stent placement compared with angiography guidance. ADAPT-DES included those with successful PCI and only those on clopidogrel and aspirin. Those with failed PCI, nonresponders to clopidogrel (by platelet function testing), and those on other P2Y12 inhibitors were excluded. The study found that IVUS guidance (compared with angiography-guidance) was associated with reduced 1-year rates of definite/probable stent thrombosis, myocardial infarction, and composite adjudicated major adverse cardiac events (ie, cardiac death, myocardial infarction, or stent thrombosis). This indicates that IVUS is a powerful tool in reducing rates of stent thrombosis and MI within 1 year after DES implantation. Limitations of this study include its observational registry design, which introduces potential for unmeasured confounding and selection bias. The results can only be applied to those with successful PCI and on clopidogrel and aspirin, rather than other platelet inhibitors. Lastly, the study population was predominantly from high-volume centers in the US and Europe, which may not reflect outcomes in lower-resource settings. Overall, the study indicates that IVUS may be a powerful tool in reducing rates of stent thrombosis and MI within 1 year after DES implantation (64).

As stent underexpansion is in an important predictor of ST and ISR, techniques such as pre- and post-dilatation have been examined to improve outcomes. Pre-dilatation refers to balloon angioplasty performed before stent deployment to facilitate stent delivery, while post-dilatation is high-pressure balloon inflation after stent placement to optimize stent expansion and apposition. The DISCO trial randomized 416 patients to direct stenting vs. stent implant following balloon pre-dilatation. Patients >75 years old, heavily calcified lesions, bifurcations, total occlusions, left main lesions and very tortuous vessels were excluded. They found that in these non-complex lesions, direct stenting without pre-dilatation is as safe and effective as stenting with pre-dilatation, with similar rates of restenosis and target lesion failure, but with reduced procedure time and radiation exposure (65). Further meta-analyses show that direct stenting modestly reduces periprocedural myocardial infarction and procedural time in simple lesions, but does not consistently lower restenosis or target vessel revascularization rates (66, 67). In complex lesions, however, the data favors using pre-dilatation. A large multicenter registry analysis of 9,525 patients demonstrated that optimal stenting technique including intracoronary imaging-guided pre-dilatation, stent sizing, and post-dilatation was associated with a significantly lower rate of cardiac events, including stent thrombosis, at 3 years in patients with complex lesions (adjusted hazard ratio for composite cardiac events 0.71, 95% CI 0.63–0.81) (68). The ACC,AHA, and SCAI recommend the use of intracoronary imaging and optimal lesion preparation, including pre-dilatation, to minimize stent thrombosis in complex PCI (69).

Although randomized controlled trials are lacking, available studies report mixed findings on the effectiveness of post-dilatation. In a *post-hoc* analysis of the BASE ACS trial, which originally studied bioactive vs. Everlimus-eluding stents, researchers examined outcomes of patients who underwent stent placement with and without post-dilatation. They found that while the rates of ISR were decreased in those who underwent post-dilatation, the rates of MACE and ST did not differ compared to those without post-dilatation. Post-dilatation was performed at the discretion of the physician rather than randomized, and were done in significantly more complex lesions (70). Furthermore, large registry data from over 90,000 stent implantations found no statistically significant difference in stent thrombosis rates between cases with and without post-dilatation, regardless of lesion complexity (71). A separate large registry of 27,148 patients from Korea examined those who underwent PCI of complex coronary artery stenosis, defined as unprotected left main disease, bifurcate lesion, diffuse-long lesion (>30 mm), or severely calcified lesions on angiography. They found that IVUS-guided post-dilation was significantly associated with a lower risk of the primary outcome (HR: 0.77; 95% CI: 0.63–0.93; *P* = 0.007), unlike post-dilation without IVUS guidance. Furthermore, compared with implanted stents, IVUS-guided post-dilation used a significantly larger post-dilation balloon (68). This suggests that IVUS-guided post-dilatation may be beneficial in complex coronary artery lesions. Further randomized trials are needed to examine the effectiveness of post-dilatation.

As discussed, bifurcation lesions have been associated with increased rates of ST compared to non-bifurcation lesions. Over the years, several bifurcation techniques have been employed to improve procedural and clinical outcomes (72). The technique descriptions are beyond the scope of this review. Multiple network meta-analyses and randomized trials consistently show that DK crush yields the lowest stent thrombosis rates among bifurcation strategies, with relative risk reductions ranging from 50% to 83% compared to provisional stenting and other two-stent methods such as culotte, T-stenting, and classic crush (73, 74). The randomized trials used in the meta-analyses did not investigate the use of this technique in more severe morbidities, such as in myocardial infarction or left ventricular dysfunction, as well as in more complex lesions such as bifurcation CTO. More studies are needed to examine the effectiveness of DK crush in these populations.

### Management of stent thrombosis

IST is a life-threatening complication for patients who have had previous stent placement, requiring urgent intervention to restore coronary perfusion. The cornerstone of management is emergent percutaneous transluminal coronary angioplasty (PTCA), which involves catheterization and balloon angioplasty to reopen the occluded stent. PTCA is often accompanied by additional stenting to help maintain the long-term patency of the lesion (78%). Concurrent antiplatelet therapy is generally recommended, with aspirin in addition to a P2Y12 inhibitor such as prasugrel, ticagrelor, or clopidogrel, to prevent further thrombotic events. Multiple studies have demonstrated >90% rates of successful reperfusion from PTCA in the setting of stent thrombosis. Despite reperfusion, IST is complicated by high rates of myocardial infarction and significant declines in left ventricular ejection fraction. Additionally, patients remain at high risk for death (11%), reinfarction (16%), and recurrent stent thrombosis (12%) in the 6 month period post emergency PTCA (75, 76). Thrombectomy may have a role in cases of higher thrombus burden, as there are lower rates of distal clot embolization and greater epicardial and myocardial reperfusion, however it has not been shown to reduce MACE (77, 78).

### Stent selection

Selecting the appropriate stent remains a significant predictor of risk of stent thrombosis. The risk of IST following PCI varies based on the type of stent used, with stent selection being made on a patient-by-patient basis. Polymer-based stents are typically the primary choice of stents used due to their safety and efficacy profiles (69). The main types of stent polymers are durable (permanent) polymers, biodegradable (bioabsorbable) polymers, and polymer-free designs. The polymer type directly influences the risk of stent thrombosis at different time periods after implantation. Durable polymer drug-eluting stents (DP-DES) commonly used include poly(ethylene-co-vinyl acetate) (PEVA), poly(n-butyl methacrylate) (PBMA), and polyvinylidene fluoride-co-hexafluoropropylene (PVDF-HFP). First-generation DP-DES are associated with a higher risk of late and very late stent thrombosis due to chronic vessel wall inflammation and delayed endothelial healing from persistent polymer presence. This risk is less pronounced with second-generation DP-DES, which use more biocompatible polymers and thinner struts, but some risk remains (79–81). Biodegradable polymer drug-eluting stents (BP-DES) are designed so the polymer degrades after drug elution, theoretically reducing chronic inflammation and the risk of very late stent thrombosis. BP-DES include polylactic acid (PLA), polyglycolic acid (PGA), poly(lactic-co-glycolic acid) (PLGA), polycaprolactone (PCL), and poly-D,L-lactide (PDLLA). Meta-analyses show BP-DES have similar rates of acute and subacute stent thrombosis compared to DP-DES, but may offer a modest reduction in very late stent thrombosis (79, 82). Polymer-free stents eliminate polymer-related inflammation entirely, but their clinical performance in terms of stent thrombosis is similar to BP-DES and second-generation DP-DES, with no clear superiority in any time period (83).

The principal alternative to polymer-based stents are the bare metal stent (BMS), which does not use any polymer or drug coating. Bare metal stents experience faster endothelialization and require shorter courses of dual antiplatelet therapy (DAPT), making them the stent of choice for patients with high risk of bleeding or with upcoming procedures that would require interruption of DAPT. BMS are associated with higher rates of restenosis compared to all drug-eluting stents (DES), regardless of polymer type, and have a higher risk of target lesion revascularization. However, BMS have a lower risk of very late stent thrombosis compared to first-generation DES, but not compared to contemporary DES. The ACC, AHA, and SCAI recommend DES over BMS for most patients due to superior efficacy and safety, including lower rates of stent thrombosis and myocardial infarction (69).

Another category of stent includes the BVS, which is designed to be completely absorbed by the body over time, including both the scaffold and any polymer or drug. Older generation BVS, such as the first-generation Absorb everolimus-eluting scaffold, were designed to provide temporary vessel support and drug delivery, then fully resorb, theoretically reducing very late adverse events associated with permanent metallic stents. Compared to second-generation EES, Absorb BVS increased the risk of device thrombosis (hazard ratio 2–4), target lesion failure, and myocardial infarction through 3 years, with the excess risk largely abating after scaffold resorption at 3 year. This pattern is consistent across large pooled analyses and individual randomized trials, including AIDA and ABSORB III/IV (84–87). The most recent clinical trial data on latest-generation bioabsorbable scaffolds with thinner struts indicate that these devices have improved safety and efficacy profiles compared to first-generation bioresorbable vascular scaffolds (BVS), but none have yet demonstrated outcomes superior to or consistently equivalent to second-generation DES such as EES, ZES, or BES in broad patient populations (84, 88–90). In summary, current clinical data do not support the use of either older or newer generation bioabsorbable scaffolds over second-generation sirolimus- or everolimus-eluting DES, which remain the standard of care for safety and efficacy (69).

### Role of antiproliferative drugs

DES are coated with antiproliferative agents, which provide both a mechanical and biochemical approach to inhibit lumen re-narrowing. The antiproliferative agents are thought to influence vessel recoil, vessel remodeling, and intimal proliferation, which are the main factors responsible for restenosis. Sirolimus and paclitaxel are the two principal antiproliferative agents used in first-generation DES. Sirolimus (a macrolide immunosuppressant) inhibits the mammalian target of rapamycin (mTOR), blocking smooth muscle cell proliferation in the G1 phase of the cell cycle. Paclitaxel (a taxane) stabilizes microtubules, arresting cell division in the G0–G1 and mitotic phases. Both drugs are delivered via durable polymer coatings on stainless steel stents in first-generation DES (e.g., Cypher for sirolimus, Taxus for paclitaxel) (doi:10.1056/NEJMra1210816, doi:10.1056/NEJMra051091). Head-to-head meta-analyses of sirolimus- and paclitaxel-eluting stents consistently show that sirolimus-eluting stents are superior to paclitaxel-eluting stents in reducing restenosis, target lesion revascularization, and stent thrombosis. Sirolimus-eluting stents (SES) are associated with a lower risk of target lesion revascularization (relative risk reduction ∼30%–40%) and a lower risk of definite or probable stent thrombosis (hazard ratio 0.66, 95% CI 0.46–0.94) compared to paclitaxel-eluting stents (PES), with no significant difference in mortality or myocardial infarction (91–93).

Second-generation DES use sirolimus analogues (everolimus, zotarolimus, biolimus A9) with more biocompatible or biodegradable polymer, further improving safety compared to first-generation SES or PES (91). A meta-analysis of 11 RCTs showed that everolimus-eluting stents (EES) had a reduced the risk of stent thrombosis compared to SES (OR: 0.44, 95% CI: 0.25–0.80) and PES (OR: 0.35, 95% CI: 0.21–0.53) (94). EES also provide lower rates of repeat revascularization and major adverse cardiac events compared to SES and PES, with no significant difference in mortality or cardiac death (95, 96). Zotarolimus-eluting stents (ZES), especially the Resolute platform, have safety profiles similar to EES, with lower stent thrombosis and myocardial infarction rates than SES and PES. Biolimus A9-eluting stents (BES) with biodegradable polymers are non-inferior to EES and ZES for efficacy, but EES and ZES remain the safest overall (97–99).

### Intravascular imaging

Intravascular imaging plays a crucial role in identifying the underlying causes of IST and guiding the appropriate interventions. Intravascular Ultrasound (IVUS) and Optical Coherence Tomography (OCT) are the primary modalities used for identifying stent-related complications such as malapposition, underexpansion, neoatherosclerosis, and quantifying thrombus burden. IVUS works through a catheter mounted ultrasound probe, providing real time cross sectional images of the coronary artery. OCT utilizes near-infrared light and contrast dye to offer a higher resolution assessment of stent morphology. IVUS has been used to identify stent underexpansion as a driver of early IST, and malapposition contributing to very late IST (100). Meanwhile, OCT's higher resolution provides superior capabilities for detecting neointimal changes and neoatherosclerosis, a major contributor to late IST (101) Intravascular imaging at the time of PCI is useful in guiding stent sizing, ensuring appropriate expansion, and detecting acute complications such as edge dissections, malapposition, and tissue protrustion (102). The RENOVATE-COMPLEX-PCI demonstrated that intravascular image guided PCI led to lower composite death from cardiac causes, target-vessel–related myocardial infarction, or clinically driven target-vessel revascularization when compared with angiographically guided PCI (103). Given these findings, the American College of Cardiology and American Heart Association recommend the use of intravascular imaging in patients with stent failure to determine the mechanism for stent failure (69).

### Duration of dual antiplatelet therapy

The decision on duration of dual antiplatelet therapy (DAPT) after PCI is dependent on the type of stent implanted, complexity of the lesion, and patient specific bleeding and thrombotic risk factorsTraditionally, bare metal stents, with their earlier endothelializationreendothelialization, require at least 30 days of DAPT (104). Previous guidelines recommended the duration of DAPT in patients with drug eluting stents as typically 6–12 months, with longer durations favored in patients presenting with ACS. although this course can be shortened based on the patient's risk factors (69). The latest generation of drug-eluting stents (DES), characterized by enhanced biocompatibility, thinner struts, and improved polymer coatings, has significantly reduced rates of stent thrombosis and myocardial infarction (69). These advancements have allowed for shorter durations of DAPT as brief as 1–3 months, in carefully selected patients, particularly those at high risk for bleeding. After this abbreviated DAPT period, transitioning to P2Y12 inhibitor monotherapy has been shown to lower major bleeding risk without increasing MACCE, compared with the traditional 12-month DAPT regimen. (doi:10.1001/jamacardio.2024.3216). In a systematic review and network meta-analysis in patients with ACS undergoing PCI 24,797 patients received either ticagrelor or prasugrel found that 1 month of DAPT followed by a P2Y12 inhibitors reduced major bleeding (RR, 0.47; 95% CrI, 0.26–0.74), however patients with 3 months of DAPT followed by PGY12 inhibitor was ranked as most optimal for reducing MACCE (RR, 0.85; 95% CrI, 0.56–1.21), despite being statically significant (69). In adition, the STOPDAPT-2 trial found that DAPT as short as one month followed by clopidogrel monotherapy may in fact be superior to the traditional regimen of 12 months of DAPT (105) Similarly, SMART-CHOICE demonstrated that P2Y12 monotherapy after 3 months of DAPT was non-inferior to 12 months of DAPT, with significantly lower bleeding risks (106). In comparison, tThe DAPT trial demonstrated that a 30 month DAPT regimen can significantly reduce the rates of stent thrombosis, however this reduction is accompanied by increased risks of bleeding and all cause mortality (61). More complex lesions are more susceptible to ischemic events such as IST. Involvement of the left main coronary artery, multiple lesions per vessel, lesions longer than 30 mm, and lesions at bifurcations are considered to be complex lesions. Given their increased ischemic risks, the ACC and AHA recommend at least 12 months of DAPT, with consideration of longer regimens based on the patient's bleeding risk assessment (107). The DAPT score is a clinical decision-making tool that can be used to stratify patients based on their bleeding and thrombotic risks and guide the duration of DAPT following coronary stent implantation. This calculator takes into account factors such as age, cigarette smoking, diabetes mellitus, myocardial infarction (MI), early-generation drug-eluting stents, stent diameter <3 mm, left ventricular ejection fraction <30%, presentation with MI, prior PCI, congestive heart failure, and vein graft PCI to identify patients who may benefit from prolonged DAPT. Conversely, a history of prior bleeding, concurrent anticoagulation therapy, female sex, advanced age, low body weight, and chronic kidney disease may place patients at higher risk for bleeding, making them better candidates for shorter DAPT regimens. According to theDAPTstudy, patients with a high DAPT score (≥2) who have tolerated 1 year of therapy without major ischemic or bleeding events are more likely to experience a favorable benefit/risk ratio with prolonged DAPT, whereas those with a low DAPT score (<2) may face increased bleeding without ischemic benefit. Thus, the DAPT score provides a practical and evidence-based approach to individualizing therapy and optimizing outcomes in patients undergoing PCI (104).

### Role of cilostazol

Cilostazol, a phosphodiesterase-3 inhibitor, has antiplatelet and vasodilatory properties, making it a potential adjunctive therapy for reducing IST after PCI. While not routinely recommended due to its contraindication in heart failure and potential adverse effects, cilostazol has been studied as part of tiple antiplatelet therapy in high-risk populations. The CILON-T trial demonstrated that cilostazol more effectively reduced platelet activity compared to standard DAPT, however it did not show a reduction in MACE after drug eluting stent implantation (108). Conversely, the CREATIVE trial found that cilostazol reduced MACE, particularly in patients with low responsiveness to clopidogrel as measured by thromboelastography, suggesting a potential role for those with resistance to clopidogrel (109). In clinical practice, cilostazol may be considered in select high risk patients, particularly those with high thrombotic risk, prior IST, or those with clopidogrel resistance (109–111). Additionally, it may be a reasonable option in those with concurrent peripheral artery disease, where its vasodilatory effects can provide additional symptomatic relief (112).

### Role of anticoagulation

In certain patient populations, the use of concurrent anticoagulation therapy and DAPT is necessary, with the most common indications being atrial fibrillation, mechanical heart valves, venous thromboembolism, and left ventricular thrombus. However, the combination of DAPT and anticoagulation (triple therapy), significant increases the risk of bleeding, with reported rates rising from 4% to 6% in DAPT to 10%–14% on triple therapy (113). Given this heightened risk, triple therapy should only be used for short durations, after which patients should be continued on anticoagulation plus a single antiplatelet agent. Studies such as the WOEST trial have demonstrated that anticoagulation combined with a single antiplatelet agent confers a lower risk of bleeding without a significant increase in thrombotic events or all-cause mortality (114, 115). Similarly, the ISAR-TRIPLE trial compared six weeks vs. six months of triple therapy, showing no significant difference in ischemic outcomes, but a reduced risk of bleeding with the shorter duration (116). Current recommendations advocate for minimizing the duration of triple therapy and transitioning to dual therapy with an anticoagulant plus single antiplatelet as soon as reasonably possible to balance thrombotic and bleeding risks effectively (117).

### Strategies for prevention

Prevention of IST requires a multifaceted approach, incorporating optimal stent selection, precise deployment, and use of intravascular imaging modalities such as IVUS and OCT to ensure proper stent expansion and apposition (102, 103). Advances in stent technology, particularly the development of second and third generation bioresorbable drug eluting stents have significantly reduced the risk of IST by improving endothelialization and reducing thrombogenicity (118, 119). Antiplatelet therapy plays a crucial role in IST prevention, with careful selection of single, dual, or triple antiplatelet therapy to balance ischemic protection with bleeding risk. The duration of antiplatelet therapy must be individualized, ranging from as short as 30 days in high bleeding risk patients, to beyond one year in those at higher risk of ischemic events (69) Adherence to the prescribed therapy is critical, as nonadherence to DAPT is associated with between a 2 and 20 fold increase in IST rates, leading to higher rates of myocardial infarction and mortality (120).

Clinical decision tools such as the DAPT score can aid in risk stratification, helping to personalize therapy duration based on the patient's unique circumstances. By integrating these strategies—careful stent selection, judicious use of intravascular imaging, tailored antiplatelet therapy, and education on medication adherence the risk of IST can be minimized, improving the long-term success of PCI.

## Limitations

Despite strong evidence and guideline recommendations to reduce stent thrombosis risk via optimal implantation techniques, imaging guidance, and prolonged dual antiplatelet therapy, adoption in real-world practice remains inconsistent. One major barrier is non-adherence, which is well documented: DAPT use often declines over time, with drop-off by 12 months, driven by bleeding, lack of education, or communication gaps (121). Cost is another recurring obstacle: newer agents (e.g., ticagrelor or prasugrel) are more expensive, and patients with lower income are less likely to adhere to antiplatelet regimens post-PCI (122). Meanwhile, imaging guidance (IVUS, OCT) offers procedural optimization and reduction in thrombosis risk, but uptake remains low. Barriers include high cost, increased procedure time, limited reimbursement, variable operator training, and institutional/regulatory constraints (123).

Beyond these classical barriers, less is known about how provider and patient preferences, and regulatory or system-level constraints, limit uptake of guidelines. Some interventionalists trained principally in angiographic guidance may resist changing habits or feel cognitive dissonance when adopting intravascular imaging (123). On the patient side, preferences regarding bleeding risk, pill burden, cost, or quality-of-life tradeoffs may lead patients to decline or discontinue recommended DAPT therapy, especially when shared decision-making is weak (https://doi.org/10.1007/s40119-024-00372-7) (124). At a higher level, variation in regulatory approval of devices/imaging modalities, institutional policies, reimbursement schemes, and the lack of enforcement of guideline adherence all impose structural friction (125).

### Future directions

When developing the next generation of stents, it is crucial to address the current limitations and side effects of current stent. As discussed in the pathophysiology section, stent placement damages the endothelium, triggering platelet activation and aggregation, which significantly increases the risk of restenosis. Therefore, advancements that minimize initial endothelial injury or enhance re-endothelialization could effectively reduce restenosis rates. Regenerative medicine, such as Re-endothelialization stents, which release therapeutic exosomes that accelerate endothelial cell proliferation and migration are one of the handful of innovations created to decrease restenosis. Re-endothelialization stents facilitate early-stage stent coverage but also reduces vascular inflammation and smooth muscle cell overgrowth, mitigating the risk of in-stent restenosis (126). However, need for further long-term studies to evaluate their clinical efficacy and safety compared to traditional stents is still needed.

Cell-captured stents use biomolecule coatings, such as antibodies targeting endothelial progenitor cells (EPCs) (e.g., anti-CD34, VE-cad, or CD133), to attract circulating EPCs and promote natural endothelial regeneration (127, 128). These stents have shown early endothelial coverage and reduced in-stent restenosis in animal models compared to BMS (129). However, clinical outcomes are inconsistent, as studies have shown that captured EPCs do not always contribute significantly to endothelial regeneration, and some cell-capture stents, like those with anti-CD34, may fail to reduce neointimal hyperplasia or even increase restenosis rates (130, 131).

Nitric oxide (NO) stents incorporate NO-producing coatings to enhance vascular healing by preventing thrombosis, reducing inflammation, inhibiting smooth muscle cell (SMC) proliferation, and promoting endothelialization (132). These stents use either NO-releasing coatings, which deliver NO from exogenous donors, or NO-generating coatings, which convert endogenous precursors into NO through catalysts like copper or selenium (133, 134). Despite promising *in vitro* and *in vivo* results, challenges include limited NO release duration, potential toxicity of NO donors, and the lack of long-term preclinical studies on NO-generating stents (135, 136).

Lastly, other research has studied VEGF-incorporated stents which utilize vascular endothelial growth factor (VEGF) coatings to promote endothelial regeneration and potentially reduce in-stent restenosis. Recent studies have shown promising results, with VEGF-bound stents selectively capturing EPCs and accelerating endothelialization (137, 138). However, outcomes remain inconsistent, as efficacy depends on co-grafting conditions, the form of VEGF, and the conjugation method (138, 139).

Nanotechnology offers significant advancements in cardiac stents by enhancing drug delivery, biocompatibility, and thrombotic pathway targeting. Liposomes and polymeric micelles facilitate controlled drug release to inhibit SMC proliferation and restenosis, while dendrimers improve gene delivery for rapid endothelial regeneration (140, 141). Lipid-polymer hybrid nanoparticles (LPNs) provide vascular targeting, reducing platelet adhesion and oxidative stress (142, 143). Nonpolymeric nanomaterials, such as TiO₂ nanotubes and diamond-like nanocomposites (DLN), enhance endothelial growth while minimizing platelet adhesion and thrombogenicity (144). These innovations improve stent hemocompatibility and reduce late in-stent restenosis, addressing key limitations of traditional DES. While nanotechnology in cardiac stents holds significant promise, it presents challenges related to toxicity and organ accumulation of nanoparticles, which can lead to adverse effects on macrophage function and increased inflammation (145, 146). Additionally, nanoparticle-induced oxidative stress and membrane disruption from materials like dendrimers and magnetic nanoparticles may contribute to cell death and toxicity, raising concerns for long-term safety in clinical applications (147). The next- generation of stents have been developed with the thought to increase biocompatibility and protect patients from restenosis in the future, but the major limitation in incorporating this new technology is the lack of longitudinal evidence supporting its safety.

### Emerging therapies targeting thrombotic pathways

Several novel drug-eluting stent (DES) technologies have been developed to improve cardiovascular outcomes and minimize thrombotic complications in patients undergoing percutaneous coronary intervention (PCI). These include Cyclopentenyl Cytosine (CPEC)-coated stents, surface-degradable DES, and stents modified through various surface engineering techniques.

CPEC-coated stents utilize an anti-proliferative nucleoside analog designed to promote endothelial cell regrowth while minimizing clot formation. By slowly releasing CPEC at the site of implantation, these stents aim to reduce neointimal tissue growth and inhibit platelet activation, ultimately improving vascular healing without the prolonged need for systemic anticoagulation. The gradual release of CPEC from the stent surface reduces neointimal hyperplasia and platelet aggregation, preventing thrombotic complications and enhancing vascular healing, as shown in preclinical models (148). However, there is a lack of human clinical trial data, so the safety, efficacy, and optimal antiplatelet regimen for CPEC-coated stents in real-world populations remain unestablished. Additionally, the long-term effects of CPEC on vascular healing and potential off-target toxicity are unknown, and the translation of findings from animal models to human coronary arteries is uncertain (148). Surface-degradable DES employ a layered coating system that incorporates a biodegradable polymer (such as PTMC) and an antiproliferative agent like rapamycin. These designs work to suppress smooth muscle cell overgrowth and reduce thrombosis while simultaneously encouraging endothelial cell recovery. The addition of an inner titanium oxide (Ti–O) layer further aids in endothelialization, contributing to improved vessel healing and decreased rates of restenosis and thrombotic events. The PTMC/rapamycin combination makes surface-degradable drug-eluting stents a promising solution for preventing restenosis and thrombosis (149). However, meta-analyses and clinical reviews indicate that while biodegradable polymer DES may reduce very late stent thrombosis compared to first-generation DES, they have not demonstrated superiority over second-generation durable polymer everolimus-eluting stents in terms of efficacy or safety. Some bioresorbable scaffolds have shown increased rates of late and very late stent thrombosis, raising concerns about their long-term safety profile (98, 150).

Additionally, advanced surface modification methods, such as plasma oxidation, physical and chemical vapor deposition (PVD/CVD), and electrodeposition—are being applied to stents to enhance their biocompatibility. These approaches alter the stent's surface characteristics, such as texture and charge, to make them less prone to clot formation. For instance, plasma treatments increase surface wettability and reduce platelet adhesion, while coatings like titanium nitride and diamond-like carbon applied via PVD or CVD have been shown to support endothelial cell adhesion and reduce thrombogenicity. Electrodeposition offers precise control over coating thickness and composition, further improving hemocompatibility and reducing thrombogenicity (151). However, there is mixed evidence regarding the impact of advanced surface modification methods on clinically meaningful endpoints such as stent thrombosis and restenosis. Many surface engineering strategies remain at the experimental or early clinical stage, with limited large-scale randomized trial data. The heterogeneity of surface modification methods and lack of standardized evaluation protocols further complicate direct comparison and clinical adoption (151, 152).

### Role of artificial intelligence

Artificial intelligence (AI) has the potential to be a valuable tool in medicine, particularly in predicting and preventing stent thrombosis (ST), but current research remains limited. Given our understanding of stent mechanics and patient risk factors, AI, specifically machine learning (ML), could be leveraged to identify individuals at higher risk for ST and tailor prevention strategies accordingly. One study by Gómez et al. (153) developed an ML model using data from the GRACIA-3 trial to stratify patient risk for stent restenosis (SR), demonstrating its potential to reduce unnecessary follow-ups and improve treatment for high-risk individuals (153). However, while ML has shown promise in pattern recognition and predictive analytics, it is only a subset of AI, true AI would likely incorporate broader reasoning, real-time decision-making, and adaptability, which could further enhance risk assessment and personalized treatment strategies.

AI-driven models, such as AI-DAPT, have demonstrated superior predictive accuracy compared to traditional risk scores by dynamically assessing ischemic and bleeding risks in post-stent implantation patients. By using vast EHR data and advanced machine learning algorithms, AI-DAPT can identify complex, nonlinear relationships among clinical variables, offering a more personalized approach to DAPT management. This adaptability is particularly relevant to stent thrombosis prevention, as AI models could continuously update risk predictions based on evolving patient data, ensuring timely intervention. As research advances, integrating AI-driven models with real-time patient data and clinical insights may provide more accurate predictions and ultimately help prevent ST. Moreover, incorporating multimodal data sources and deep learning techniques could further refine risk assessment, improving long-term cardiovascular outcomes (154, 155). However, the clinical utility of AI in this domain is still developing, necessitating further validation through large-scale studies and real-world implementation.

## Conclusion

ST remains a complex and multifactorial complication following PCI, influenced by patient-specific characteristics, procedural factors, lesion complexity, and underlying medical co-morbidities. Advanced age, female sex, smoking, and obesity are notable patient-related risk factors that increase thrombosis risk. These factors, alongside nonadherence to DAPT, remain significant modifiable risks, especially in the early post-PCI period. Furthermore, procedural factors like stent under-expansion, malposition, edge dissections, and improper stent sizing necessitate the use of advanced intravascular imaging techniques, such as intravascular ultrasound (IVUS) and optical coherence tomography (OCT), which can optimize stent placement and reduce complications. Complex lesion characteristics, including bifurcations, calcifications, chronic total occlusions (CTOs), and in-stent restenosis, increase the risk of thrombosis and highlight the need for tailored interventional strategies. In addition, chronic conditions such as diabetes, chronic kidney disease (CKD), and hypercoagulable states exacerbate thrombotic risk through mechanisms like endothelial dysfunction and impaired arterial healing. Studies have shown that diabetes accelerates atherosclerosis and impairs stent healing, leading to higher rates of late and very late ST. Given the interplay of these risk factors, a comprehensive, multidisciplinary approach is essential to minimize the incidence of ST and improve long-term patient outcomes. This approach must include individualized procedural techniques, medical management tailored to the patient's unique risk factors, and rigorous adherence to DAPT.

The pathophysiology of ST is multifaceted, driven by platelet activation, delayed endothelialization, and hemodynamic factors. Studies have shown that first-generation drug-eluting stents (DES), such as paclitaxel-eluting stents, cause heightened platelet activation due to their polymeric surfaces, while second-generation cobalt-chromium DES exacerbate thrombin generation. Delayed endothelialization, particularly with sirolimus- and paclitaxel-eluting stents, prolongs stent exposure to circulating blood elements, sustaining a prothrombotic state. Additionally, hemodynamic alterations, such as endothelial shear stress, stent mispositioning, and strut design, contribute significantly to thrombogenicity by promoting platelet adhesion and thrombus formation. Stent misalignment and thicker struts exacerbate low-shear environments conducive to thrombus development, underscoring the need for precise stent placement. These findings further reinforce the importance of continued advancements in stent technology and implantation techniques to mitigate thrombosis risk. Given these complex interactions, multidisciplinary management, incorporating cardiologists, interventional specialists, and pharmacologists, is crucial to ensure that all aspects of patient care, from procedural optimization to pharmacological intervention and long-term management, are addressed. Multimodal strategies that integrate state-of-the-art imaging, personalized treatment plans, and adherence to evidence-based therapies are critical for improving patient outcomes and reducing ST incidence. Despite advances in stent technology and pharmacotherapy, several challenges remain in minimizing the incidence of ST. There is an urgent need for further research to better understand the role of specific lesion characteristics, stent designs, and patient risk factors in stent thrombosis development. Clinical trials, such as those by Palmerini et al. (63) and studies involving advanced intravascular imaging techniques, should continue to focus on optimizing procedural strategies and stent material innovation (63). Additionally, research into new therapeutic agents, such as those targeting thrombotic pathways and advancements in nanotechnology for stent coatings, holds promise for enhancing endothelial healing and reducing thrombosis risk.

As stent thrombosis remains a critical issue in PCI outcomes, a continued, multidisciplinary effort combining clinical expertise, technological innovation, and patient-centered care is essential to improving the prevention, diagnosis, and management of this complication.
